# Analysis of Shock Wave Interaction with an Obstacle by Coupling Pressure Measurements and Visualization

**DOI:** 10.3390/s22093325

**Published:** 2022-04-26

**Authors:** Antoine Gautier, Isabelle Sochet, Sébastien Courtiaud

**Affiliations:** 1 Laboratoire PRISME, INSA Centre Val de Loire, F-18020 Bourges, France; antoine.gautier@insa-cvl.fr; 2 CEA, DAM, CEA-Gramat, F-46500 Gramat, France; sebastien.courtiaud@cea.fr

**Keywords:** shock wave, experiments, pressure sensor, visualization, correlation, Mach stem

## Abstract

Small-scale experiments are a good means of carrying out explosion and shock wave measurements. Commonly, the shock wave is tracked thanks to pressure sensors and sometimes with a high-speed camera. In the present study, these methods were used to analyze the interaction of a shock wave with an obstacle of simple geometry. The primary aim of the study was to demonstrate the need to correlate these different methods in order to analyze certain phenomena related to the three-dimensional interaction of a shock wave with an object. The correlation between the overpressure and the visualization made it possible to carry out a complex analysis. The visualization was carried out simultaneously on two planes, from the front and top views, thanks to the optical setup. Shock wave characteristics were taken at ground level downstream of the obstacle with pressure gauges. The correlation of the images obtained allows the identification of the waves on the profile and their contribution in intensity.

## 1. Introduction

The study of the effects produced by an explosion is an expanding field of research in the world of defense and that of industrial risks. The terrorism attack in Oslo, 22 July 2001, killed 8 people and injured 15 others during the first bomb explosion. Eight suicide bombers in the attacks in Sri Lanka caused the death of more than 320 people. The explosion of the AZF factory (21 September 2001, Toulouse, France) caused extensive damage, the death of 31 people and injuries to 2500 others [1]. More recently, on 4 August 2020, the harbor of Beirut suffered two explosions, resulting in 207 deaths and 6500 injuries. If the damage in these two examples was so extensive, it is partly because of the shock wave produced by the explosion, which covered over several kilometers in urban areas [2,3,4].

Even today, however, these studies require an experimental approach, as the phenomena encountered are highly complex and still need to be explored. Small-scale tests have now proved their worth in terms of both cost and quality of results, safety, and reproducibility of test conditions. With small-scale experiments, it is easy to conduct parametric studies which are useful to validate numerical simulations [5,6,7,8,9,10].

The tracking of a shock wave is commonly conducted using two different experimental means: with pressure sensors or with a high-speed camera. The pressure sensors provide the temporal and local evolution of the overpressure. This makes it possible to determine the value of the maximum overpressure and impulse produced by any shock wave that passes over the pressure sensor. The camera provides primarily qualitative information on the structural evolution of shock waves. The optical model of Edgerton [11] is the typical method used to visualize shock waves in a large field. Nevertheless, some optical setups make it possible to quantify the shock wave at the cost of some optical constraints or corrections [12,13,14,15,16]. A simple optical method is the pure in-line shadowscopy (PILS) method, also called retroreflective Edgerton shadowgraphy. In this method, the optics are installed in such a way that the main axis of the camera and the axis of the projected light coincide. The simultaneous realization of these two means of measurement provides complementary and synchronized information, allowing for a more in-depth analysis of the phenomena [17]. Thanks to pressure sensors situated in the visualized axis, Maillot et al. [17] showed a correlation between the Mach stem formation downstream of a semi-infinite obstacle and the increase in the maximum overpressure. In their study, Maillot et al. [17] used a high-speed camera with the optical method PILS.

The purpose of this paper is to present a more complete method for analyzing the interaction of a shock wave with an obstacle. This analysis is based on measurements of the shock wave overpressure downstream of an obstacle with the support of the visualization in front view and top view. The experimental bench and obstacles are described in the following section. In Section 3, the shock wave interaction with an obstacle is analyzed, using the pressure sensors to quantify the shock wave and visualization for qualitative information. In Section 4, only the visualization is used to carry out measurements on the shock waves.

## 2. Materials and Methods

### 2.1. Experimental Bench

**Figure 1:**
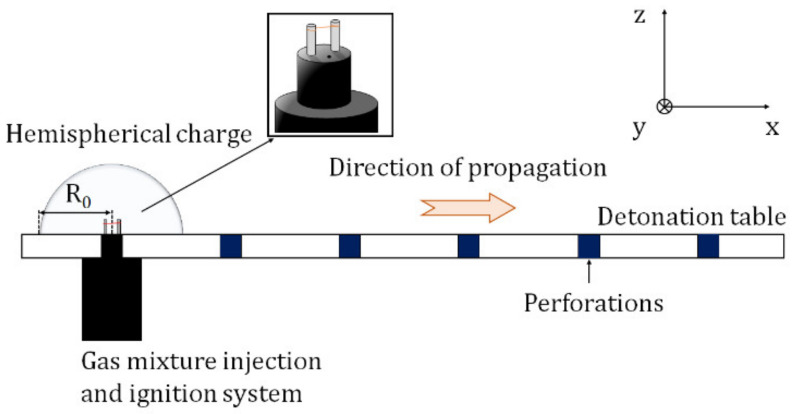
Detonation table and ignition device.

The shock wave is produced by detonating a stoichiometric gaseous mixture of propane-oxygen [18,19]. The gaseous charge is confined in a hemispherical soap bubble with a radius of 60 mm. Hence, the mass of the explosive charge m_c_ = 6.2882 × 10^−4^ kg. Ignition is created by discharging instantaneously a high voltage (8 kV in 1 μs) to sublimate a tinned copper wire between two electrodes. Figure 1 shows a diagram of the detonation table and the ignition device. Here, one can see that the two electrodes are in the charge center. This diagram represents a free field configuration, i.e., only the explosive charge is on the detonation table, without any obstruction.

As Figure 1 shows, the detonation table is perforated in order to lay out the pressure sensors. The pressure sensors are embedded in an absorbent material (Nylatron plastic or polyamide) in order to attenuate the vibrations of the table. The pressure sensors are piezoelectric sensors PCB113B26 and transmit a voltage signal with a rise time ≤1 µs. The constructor defined the rise time in shock tube and the sensor is in the reflected position. The signals then pass through an amplifier before being transmitted to the data acquisition system with a range of 10 V. The data acquisition system is a Dewetron DW-801 with 16 channels and a sampling rate of 1 MHz/channel independently of the acquisition time. Thanks to its large storage capacity, this system allows long periods of pressure acquisition, while maintaining maximum signal quality. However, in order to avoid storing unnecessary data, only 5 ms are recorded after the explosion is initiated, which is more than necessary to measure the passage of the shock wave in our configurations. All information about pressure sensor properties and the filter used is detailed in Table 1.

**Figure 2:**
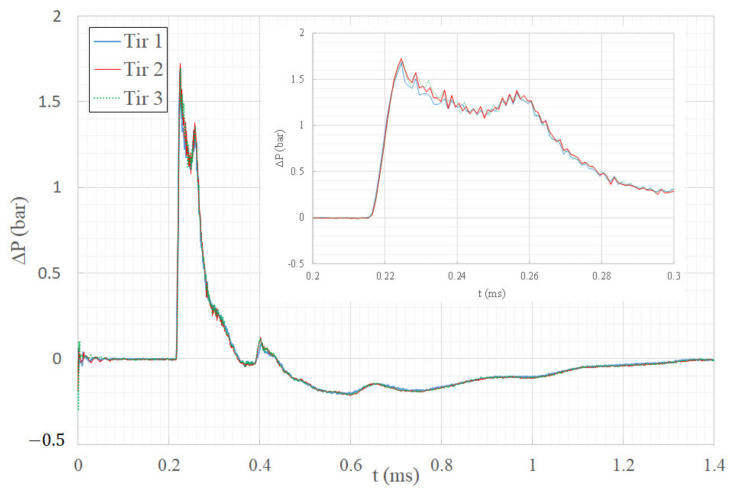
Overpressure time history in free field.

Three overpressure profiles measured 203 mm from the center of the explosion in free field to the center of the sensor surface are illustrated in Figure 2. These three curves are quite similar and show the repeatability of experiments with a tolerance threshold below 5%. These small-scale experiments were compared with a nominal 20-ton propane/oxygen explosion [20], and the excellent agreement indicates the validity of Hopkinson’s cube-root scaling applied to propane/oxygen explosions over a range of charge masses in excess of six orders of magnitude.

The pressure measurements were complemented by the visualization. The visualizations were performed using the pure in-line shadowscopy (PILS) method. This method is well-adapted to the study of shock waves due to the simplicity and high performance of the setup [21]. In this setup, the divergence of the light source makes it possible to visualize a large area (a few tens of centimeters). In addition, the axis of the light source coincides with the viewing axis of the high-speed camera, allowing quantifiable observations. The light source is an LED lamp Bridgelux 50C10K1-C-7, which emits a luminous flux of 10,000 lumen. The light is emitted perpendicularly to the axis of the fast camera. The light rays are concentrated by a plano-convex lens with a focal length of 85 mm towards a 45° beveled mirror located in the middle of the camera lens.

**Figure 3:**
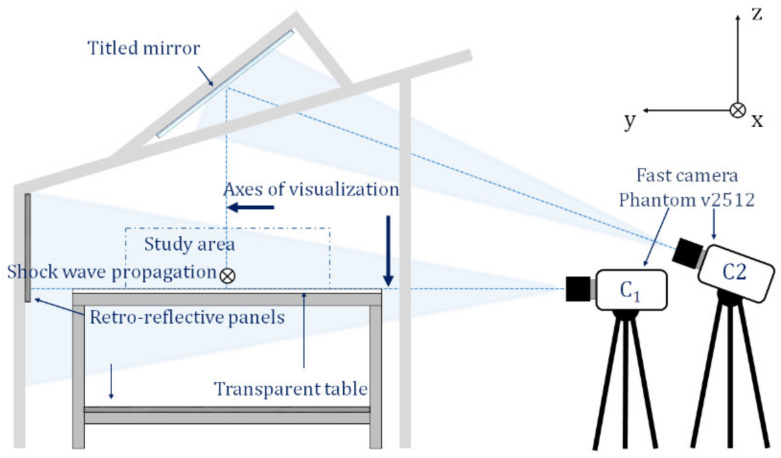
Optical installations.

A particularity of the present optical bench is that two fast Phantom v2512 cameras were used. Both of the cameras have a resolution of 640 × 480 pixels with a sample rate of 70,000 fps and are triggered at the same time. The first camera, called C_1_, records the horizontal propagation of the shock wave, while the second camera, called C_2_, records the shock wave propagation in the vertical direction thanks to a tilted mirror. The surface of the mirror is 1 m^2^ and is inclined about 34° from the horizontal direction. The cameras are focused to observe the shock wave propagation along the axis that passes through the center of the explosive charge and the center of the obstacle. Figure 3 illustrates the optical installations. The recorded images were processed using the open-source software ImageJ. The processing method used is background subtraction.

### 2.2. Obstacle Configuration

There are two obstacle configurations. Each configuration corresponds to one obstacle shape. The first obstacle is a cylinder, 111 mm in diameter and 92 mm in height, called Cyl-111-92. The second one is a rectangular parallelepiped with a square section of 45 mm × 45 mm and a length of 92 mm, called Parall-45-92-45. These two obstacles are considered to be totally rigid and reflective. The center of the obstacle is situated 473 mm from the charge center.

## 3. Analysis by Coupling Pressure Measurements and Visualization

In this part, the shock wave interacts with the cylindrical obstacle Cyl-111-92. For this configuration, the experiments were performed twice. The first time, only the visualization was carried out in order to observe the shock wave propagation under optimal conditions, since the presence of pressure sensors on the detonation table disturbs the visualized field. Then, the experiments were performed again, this time measuring only the overpressure. This was possible only thanks to the good repeatability of the tests.

**Figure 4:**
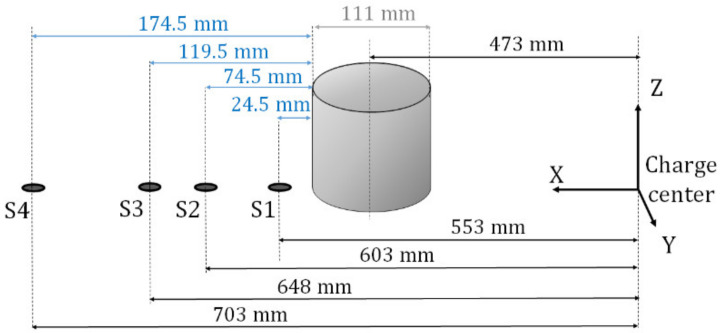
Layout of the pressure sensors downstream of the cylinder.

Four pressure sensors are situated downstream of the cylinder as illustrated in Figure 4. The distances are taken from the center of the charge or the downstream cylinder to the center of the sensor surface.

In both experiments, all pressure sensors are in the field of view of the two cameras, except the pressure sensor S4.

**Figure 5:**
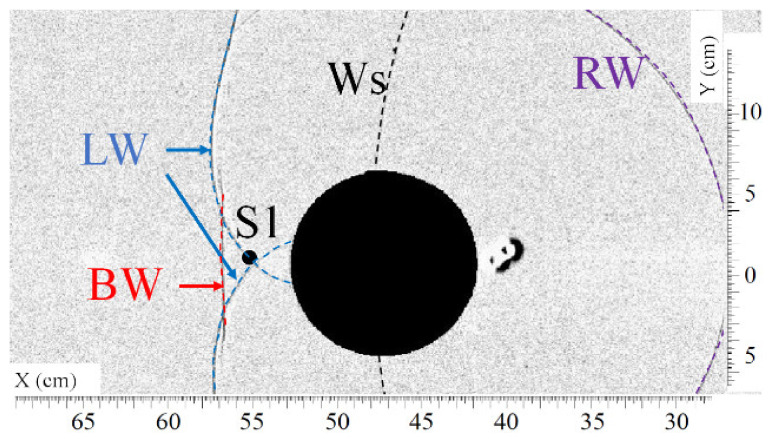
Visualization of the shock waves (LW) passing approximately over sensor S1 at t = 1.100 ms. (Ws) secondary shock wave, (RW) reflected shock wave, (LW) lateral shock wave, (BW) bypass shock wave.

**Figure 6:**
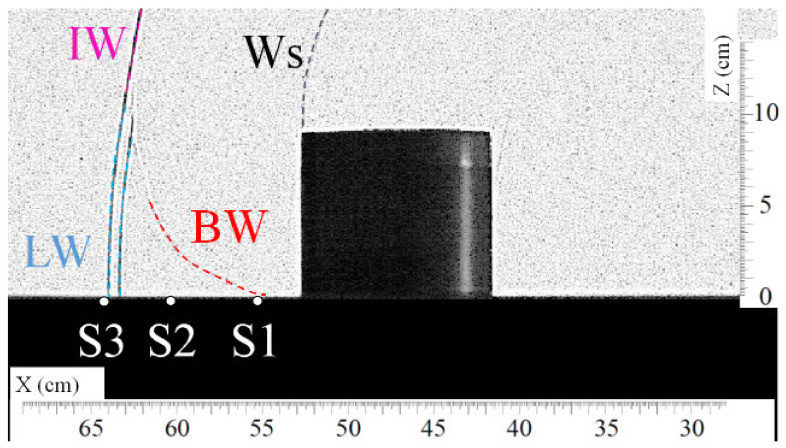
Visualization of the shock wave (BW) passing approximately over sensor S1 at t = 1.257 ms. (Ws) secondary shock wave, (IW) incident shock wave, (LW) lateral shock wave, (BW) bypass shock wave.

**Figure 7:**
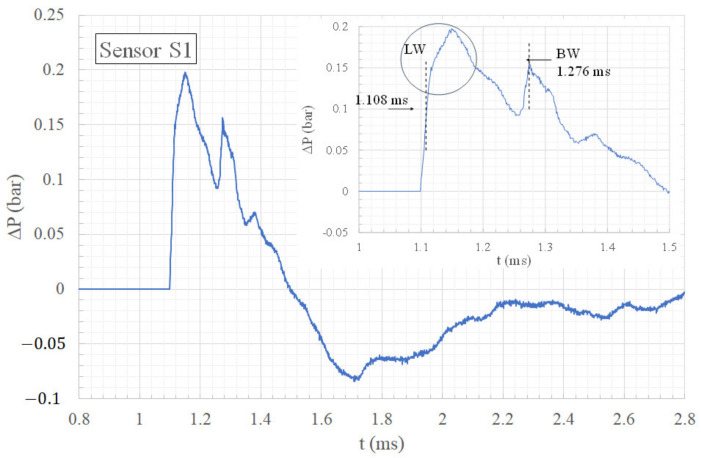
Overpressure time history of sensor S1.

Figure 5 and Figure 6 show, respectively, the top view and the front view of the shock wave as it passes approximately over sensor S1. In Figure 5, one can observe the intersection of the lateral waves (LW) downstream of the cylinder at the position of sensor S1 at the time t = 1.100 ms. The overpressure profile of sensor S1 plotted in Figure 7 shows the arrival of the shock waves. The origin of the overpressure peaks is known thanks to the visualizations. The slope of the curve at the top of the first overpressure peak (circled portion) is weak compared to the slope of the curve in free field, as seen in Figure 2. This is certainly due to an imperfect alignment of the sensor with the cylinder, which implies that the crossing point of the lateral waves (LW) does not pass exactly over sensor S1.

Figure 6 is a front visualization that shows the diffraction of the shock wave on the top of the cylinder at t = 1.257 ms. At this moment, the bypass wave (BW) reaches the ground at the position of sensor S1. One can observe the two lateral wave fronts because of the parallax.

Thanks to the visualization illustrated in Figure 6, it can be deduced that the second overpressure peak at t = 1.276 ms in Figure 7 is produced by the bypass wave (BW). The wave (BW) reaches sensor S1 0.168 ms later than the lateral waves (LW), so that its overpressure occurs during the decreasing phase of the overpressure produced by the lateral waves (LW). At the passage of the wave (BW), the overpressure is 20% weaker than the maximum overpressure.

**Figure 8:**
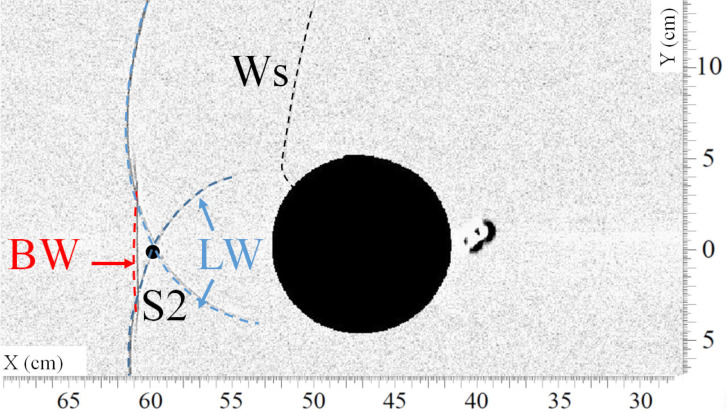
Visualization of the shock waves (LW) passing approximately over sensor S2 at t = 1.214 ms. (Ws) secondary shock wave, (LW) lateral shock wave, (BW) bypass shock wave.

**Figure 9:**
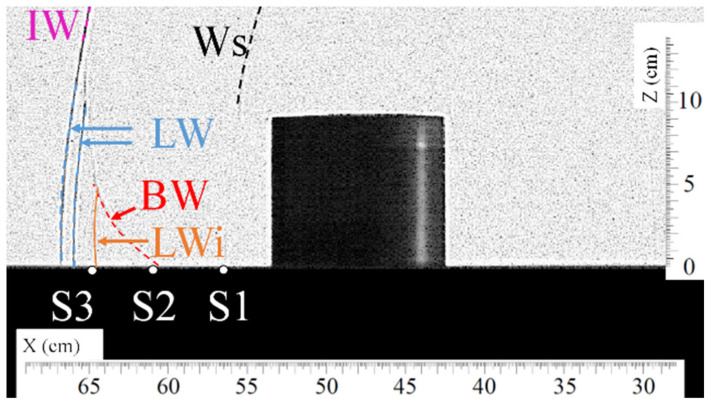
Visualization of the shock wave (BW) passing approximately over sensor S2 at t = 1.314 ms. (Ws) secondary shock wave, (IW) incident shock wave, (LW) lateral shock wave, (BW) bypass shock wave, (LWi) intersection of lateral shock waves.

**Figure 10:**
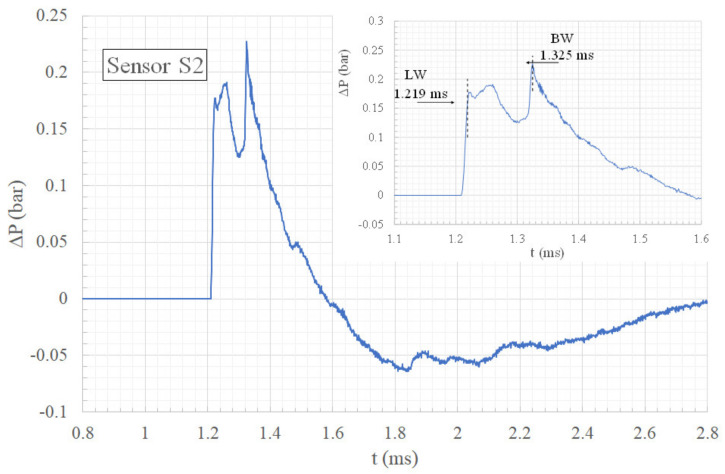
Overpressure time history of sensor S2.

Figure 8 and Figure 9 present, respectively, a top view and a front view of the shock wave when it reaches approximately sensor S2. Figure 10 gives the overpressure measured by sensor S2. In Figure 8, the lateral waves (LW) still intersect in one point situated over sensor S2 at t =1.214 ms. Around 0.1 ms later, the bypass wave (BW) reaches the position of sensor S2, as can be seen in Figure 9. In this picture, one can also notice that a new wave is visible, called LWi. Wave LWi is the intersection of the two lateral waves.

This time, the overpressure evolution plotted in Figure 10 shows that the maximum overpressure is produced by the bypass wave (BW). It is 21% higher than the maximum overpressure produced by the lateral waves (LW). The curve also shows that the bypass wave (BW) arrives 106 ms later than the lateral waves (LW). Therefore, comparing this delay with the one measured at sensor S1, one can observe that the wave (BW) takes 0.062 ms less than the lateral waves (LW) to travel the same distance between S1 and S2. That means that the bypass wave (BW) moves faster than the lateral waves (LW). This is due to the fact that the bypass wave (BW) propagates in the medium just shocked by the lateral waves (LW). Moreover, the wave (BW) gains in intensity with distance.

**Figure 11:**
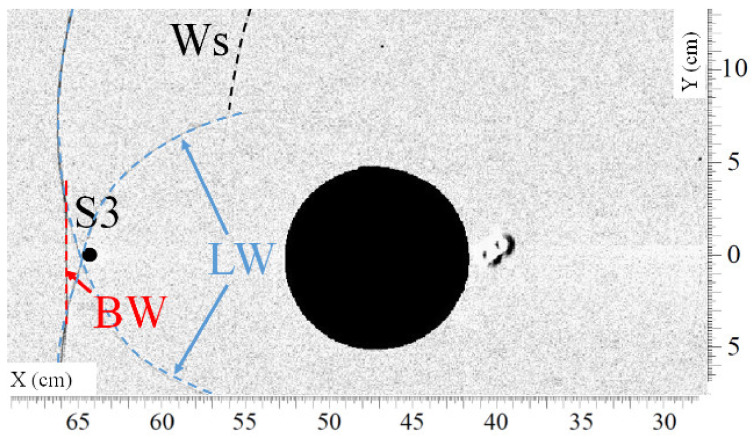
Visualization of the shock waves (LW) passing approximately over sensor S3 at t = 1.342 ms. (Ws) secondary shock wave, (LW) lateral shock wave, (BW) bypass shock wave.

**Figure 12:**
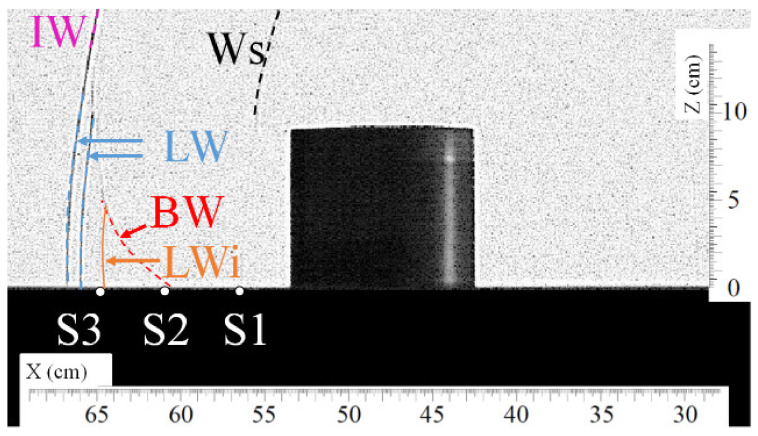
Visualization of the shock wave (BW) passing approximately over sensor S3 at t = 1.399 ms. (Ws) secondary shock wave, (LW) lateral shock wave, (BW) bypass shock wave, (LWi) intersection of lateral shock waves.

**Figure 13:**
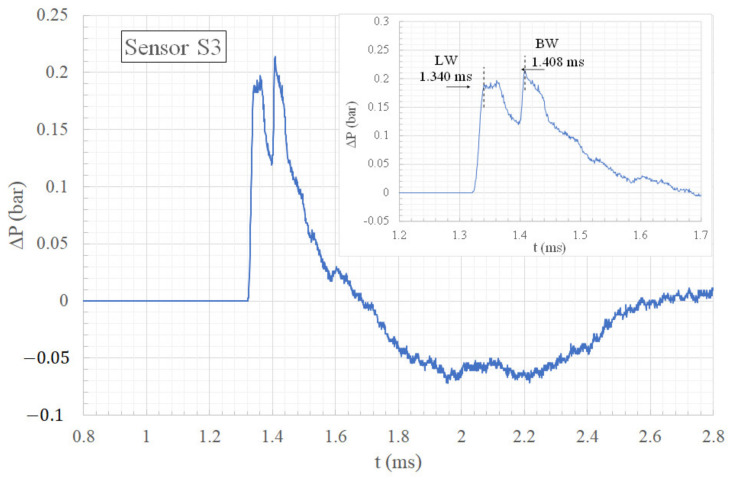
Overpressure time history of sensor S3.

Visualizations of the passage of the different shock waves at the level of sensor S3 are presented in Figure 11 and Figure 12. The corresponding pressure profile is illustrated in Figure 13. Here, one can see that the bypass wave (BW) reaches sensor S3 0.068 ms later than the lateral waves (LW). Again, the maximum overpressure is caused by the second impulse produced by the bypass wave (BW) arriving very quickly after the lateral waves (LW). The maximum overpressure is then increased by 13% compared to the maximum overpressure produced by the lateral waves (LW).

From this study coupling measurements of pressure and visualization, it has been demonstrated that the complementarity of the data can be necessary to know the origins of phenomena. For instance, thanks to the visualization, it was observed that the second overpressure peak is caused by the bypass wave (BW).

## 4. Quantitative Measurements by Visualization

In this part, only the visualization is considered. As already mentioned, the optical method used is the method PILS, which allows quantitative measurements, such as the shock wave velocity or the trajectory of the triple point over a large distance. The shock wave is observed interacting with the parallelepiped object Parall-45-92-45. The viewing area with camera C1 is 449 × 337 mm^2^ and, with camera C2, 461 × 346 mm^2^.

**Figure 14:**
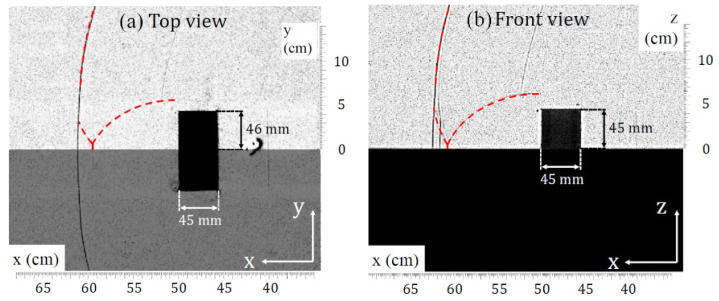
Comparison of the bypass wave and the lateral waves downstream of the obstacle Parall-45-92-45. (**a**) Top view and (**b**) front view.

In this configuration, the obstacle is oriented so that its greatest length is perpendicular to the direction of propagation of the shock wave (i.e., horizontal plane). Figure 14 presents a visualization of the interaction of the shock wave with the obstacle Parall-45-92-45.

The interest of the obstacle Parall-45-92-45 is that its height (45 mm) is almost half its length (92 mm). Therefore, the distance traveled by the diffracted wave around the top face of the obstacle can be considered equal to that traveled by the diffracted wave around the lateral faces of the obstacle. In Figure 14a, the top view shows the interaction of the lateral waves. This interaction is considered as an ideal reflection, as suggested by Dewey et al. [12], i.e., there is no loss of energy. Here, the interaction of the lateral waves produces a Mach stem in the plane (xy). In Figure 14b, the front view shows the bypass wave and its reflection on the ground downstream of the obstacle. In line with Dewey et al. [12], this reflection is considered as a real reflection. An irregular reflection occurs and a Mach stem forms in the plane (xz). Therefore, one can observe that a Mach stem appears in both planes (xz) and (xy).

**Figure 15:**
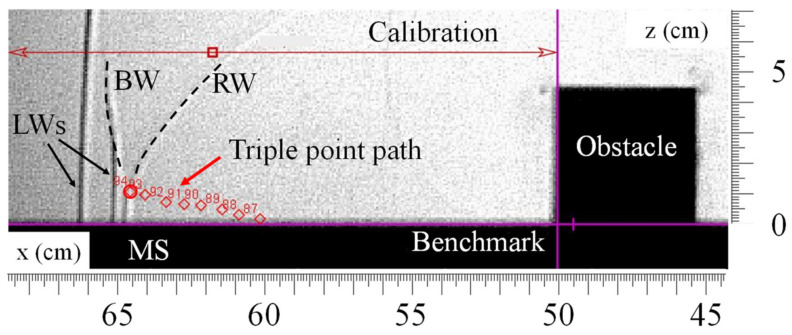
Tracking of the triple-point path on Tracker.

These Mach stems can be measured with tracking software, such as the open-source Tracker. The way measurements are carried out is illustrated in Figure 15.

The benchmark is centered downstream of the obstacle so that the triple-point path can be expressed from this position. The triple-point path measured downstream of the obstacle was compared to models from the literature. Nevertheless, among the different models studied [12,13,14,15,16], only the model of Kinney and Graham [12] was considered relevant because it does not depend on the explosive charge mass, but only on the Mach number of the incident shock wave. In the literature, some models [22,23,24,25,26] suggest an approximation of the Mach stem evolution following an explosion above the ground. This configuration is also called height of burst (HOB). In this case, the incident shock wave produced by the explosion interacts with the ground and is reflected. Initially regular, the reflection becomes irregular under certain conditions, especially depending on the reflected wave intensity and its angle against the ground.

**Figure 16:**
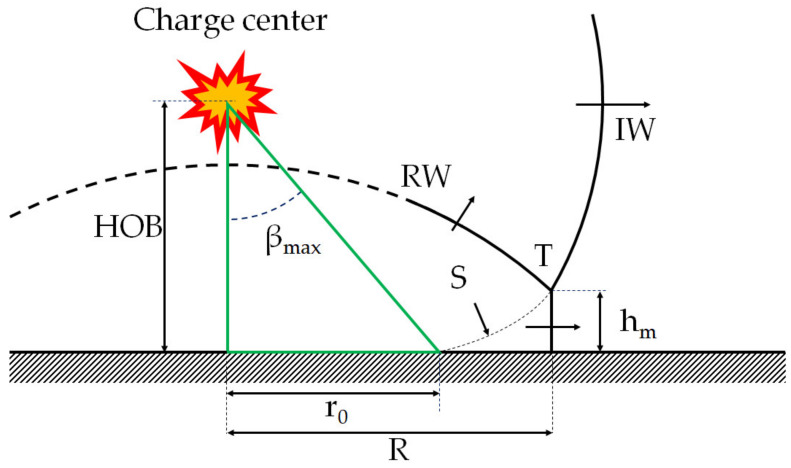
HOB and formation of the Mach stem. (IW) incident wave, (RW) reflected wave, T triple point, S slipstream.

In the present study, the Mach stem comes from the reflection of the diffracted wave downstream of the obstacle. The intensity of the diffracted wave is lower than that of the incident wave. Therefore, it is inappropriate to use models which depend on the explosive charge mass. Among the different models studied [22,23,24,25,26], only the model of Kinney and Graham [22] was considered relevant because it depends only on the Mach number of the incident shock wave. An illustration of the formation of the Mach stem in the case of an explosion above the ground, the HOB configuration, is given in Figure 16. The angle β_max_ is formed by the position of the transition distance r_0_ between regular reflection and irregular reflection with Mach stem formation.

According to Kinney and Graham [22], the maximum angle β_max_ is given by:

(1) $$\beta_{\max}\left({}^{\circ}\right)\;=\;\frac{1.75}{M_{0}\;-\;1}\;+\;39$$

From Equation (1), the position of the transition between regular reflection and irregular reflection r_0_ can be expressed as follows:

(2) $$r_{0}\;=\;\mathrm{HOB}\tan\left(\beta_{\max}\right)$$

Finally, the Mach stem height h_m_ equation is given by:

(3) $$h_{m}\;=\;0.07\;\mathrm{HOB}{\left(\frac{R}{r_{0}}\;-\;1\right)}^{2}$$

To be able to compare the triple-point trajectory downstream of an obstacle to a model of triple-point trajectory in an HOB configuration, it is considered that the HOB is equal to the obstacle height.

**Figure 17:**
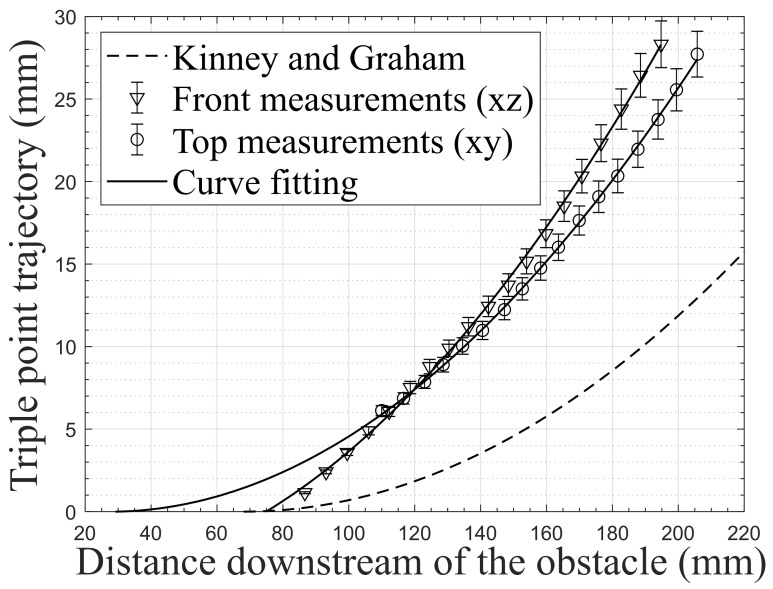
Triple-point trajectory measured downstream of the obstacle Parall-45-92-45 in both directions.

Figure 17 shows the triple-point trajectory downstream of the obstacle Parall-45-92-45 in both directions and the theoretical path from the model of Kinney and Graham [22]. This is mainly due to the fact that the center of curvature of the diffracted wave BW is not exactly the height of the obstacle. A curve fitting the measured points is used with the Matlab tool to determine the appearance of the triple point. The curves are second-degree polynomials, as is the case in all triple-point trajectory models.

From Figure 17, it appears that the model of Kinney and Graham does not fit with measurements, except for the appearance of the triple point measured with the front view. This is mainly due to the fact that the center of curvature of the shock wave is not exactly the height of the obstacle. Regarding the evolution of the Mach stem height in both directions, it is interesting to see that it does not appear in the same position and that the curves are not parallel, as expected from the findings of Dewey et al. [12]. This can be explained by the fact that the lateral waves (LW) do not only interact with each other, but also with the bypass wave (BW).

To conclude in this part, the use of visualization in both directions to generate a front view and a top view can be a real asset to analyze the interaction of a shock wave with an obstacle. In a three-dimensional experiment, the shock waves interacting with an obstacle are not observable in the same plane. The setup presented here is of real interest for the understanding of shock waves and their propagation in a complex medium.

## 5. Conclusions

In the present study, experiments to analyze the interaction of a shock wave with an obstacle have been presented. The experiments were performed on a small scale in an open field with a gaseous explosive charge. The aim of this paper was to highlight the use of co-ordinate cameras in two directions (front view and top view) associated with the measurements of overpressure, since the interaction of a shock wave with a finite object produces three-dimensional effects that cannot be seen with only one camera or only with the overpressure measurements. Two examples have been presented. In the first example, the shock wave interacted with a cylinder. The use of two cameras associated with the pressure sensors made it possible to determine the origin of the maximum overpressure. Then, in the second example, the shock wave interacted with a parallelepiped. Using the two cameras in both directions enabled us to compare the Mach stem formed by the interaction of the bypass wave with the ground and that formed by the interaction of the two lateral waves.

## Figures and Tables

**Table 1:** Pressure sensor PCB 113B26 information from specification document PCB (https://www.pcb.com/contentStore/docs/pcb_corporate/pressure/products/specsheets/113b26_d.pdf, accessed on 10 April 2022).

Sensor Name	Sensitivity (V/bar)	Uncertainty Specific to the Calibration (%)	Pressure Measurement Capacity Range (bar)	Filter Type	Filter Prototype
S1	0.1386	±1.3	0–34.4	Low pass	Butterworth
S2	0.1396				
S3	0.1443				
S4	0.1408				

## Data Availability

Not applicable.

## References

[1] Dechy N. (2004). The damage of the Toulouse disaster, 21 September 2001. Loss Prev. Bull..

[2] Rigby S.E., Lodge T.J., Alotaibi S., Barr A.D., Clarke S.D., Langdon G.S., Tyas A. (2020). Preliminary yield estimation of the 2020 Beirut explosion using video footage from social media. Shock Waves.

[3] Aouad C.J., Chemissany W., Mazzali P., Temsah Y., Jahami A. (2021). Beirut explosion: TNT equivalence from the fireball evolution in the first 170 milliseconds. Shock Waves.

[4] Dewey J.M. (2021). The TNT and ANFO equivalences of the Beirut explosion. Shock Waves.

[5] Reichenbach H., Neuwald P., Kuhl A.L. Role of Precision Laboratory Experiments in the Understanding of Large-Scale Blast Phenomena, Julius J. Meszaros Lecture. Proceedings of the International Symposium on Military Aspects of Blast and Shock (MABS17).

[6] Neuwald P., Reichenbach H. Detonations in Front of a Tunnel Entrance: A Parametric Small-Scale Study. Proceedings of the International Symposium on Military Aspects of Blast and Shock (MABS17).

[7] Smith P.D., Rose T.A., Ng S.H. The influence of areal density on the shielding and channeling of blast by buildings. Proceedings of the International Symposium on Military Aspects of Blast and Shock (MABS18).

[8] Ripley R.C., Von Rosen B., Ritzel D.V., Whitehouse D.R. Small-scale modeling of explosive blasts in urban scenarios. Proceedings of the 21st International Symposium on Ballistics.

[9] Miura A., Matsuo A., Mizukaki T., Shiraishi T., Utsunomiya G., Takayama K., Nojiri I. (2004). Reflection and Diffraction Phenomena of Blast Wave Propagation in Nuclear Fuel Cycle Facility. Jpn. Soc. Mech. Eng..

[10] Rose T.A., Smith P.D., May J.H. (2006). The interaction of oblique blast waves with buildings. Shock Waves.

[11] Edgerton H.E. (1958). Shock Wave Photography of Large Subjects in Daylight. Rev. Sci. Instrum..

[12] Dewey J.M., Mcmillin D.J., Classen D.F. (1977). Photogrammetry of spherical shocks reflected from real and ideal surfaces. J. Fluid Mech..

[13] Kleine H., Timofeev E., Takayama K. (2005). Laboratory-scale blast wave phenomena—Optical diagnostics and applications. Shock Waves.

[14] Hargather M.J., Settles G.S. (2007). Optical measurement and scaling of blasts from gram-range explosive charges. Shock Waves.

[15] Hargather M.J., Settles G.S. (2009). Retroreflective shadowgraph technique for large-scale flow visualization. Appl. Opt..

[16] Hargather M.J. (2013). Background-oriented schlieren diagnostics for large-scale explosive testing. Shock Waves.

[17] Maillot Y., Sochet I., Vinçont J.-Y., Grillon Y. Experimental evaluation of a shock wave propagation impacting surface irregularity. Proceedings of the International Symposium on Military Aspects of Blast and Shock (MABS25).

[18] Sochet I., Sauvan P., Boulanger R., Nozeres F. (2014). External explosion in an industrial site. J. Loss Prev. Process. Ind..

[19] Gault K., Sochet I., Hakenholz L., Collignon A. (2020). Influence of the explosion center on shock wave propagation in a confined room. Shock Waves.

[20] Dewey J.M., Sochet I. (2021). Analysis of the blast waves from the explosions of stoichiometric, rich, and lean propane/oxygen mixtures. Shock Waves.

[21] Slangen P., Lauret P., Aprin L., Heymes F., Lecysyn N. Optical characterizations of falling droplets interacting with shock wave. Proceedings of the 10th Pacific Symposium on Flow Visualization and Image Processing (PSFVIP10).

[22] Kinney G.F., Graham K.J. (1985). Explosive Shocks in Air.

[23] McKinzie M.G., Cochran T.B., Norris R.S., Arkin W.M. (2001). The US Nuclear War Plan: A Time for Change.

[24] U.S. Department of the Army, Unified Facilities Criteria (UFC) (2008). Structures to Resist the Effects of Accidental Explosions.

[25] Boutillier J., Ehrhardt L., De Mezzo S., Deck C., Magnan P., Naz P., Willinger R. (2017). Evaluation of the existing triple point path models with new experimental data: Proposal of an original empirical formulation. Shock Waves.

[26] Xiao W., Andrae M., Gebbeken N. (2020). Development of a new empirical formula for prediction of triple point path. Shock Waves.

